# ZNF692 drives malignant development of hepatocellular carcinoma cells by promoting ALDOA-dependent glycolysis

**DOI:** 10.1007/s10142-024-01326-x

**Published:** 2024-03-08

**Authors:** Weiwei Meng, Xiaojuan Lu, Guanglei Wang, Qingyu Xiao, Jing Gao

**Affiliations:** 1 Department of Laboratory, Shenzhen Baoan Shiyan People’s Hospital, No. 11, Jixiang Road, Shiyan Street, Baoan District, Shenzhen, Guangdong 518108 P.R. China; 2 Department of Blood Transfusion, Shenzhen Baoan Shiyan People’s Hospital, Shenzhen, Guangdong 518108 P.R. China

**Keywords:** Hepatocellular carcinoma, Glycolysis, ZNF692, ALDOA, Acetylation

## Abstract

Hepatocellular carcinoma (HCC) is one of the malignancies with the worst prognosis worldwide, in the occurrence and development of which glycolysis plays a central role. This study uncovered a mechanism by which ZNF692 regulates ALDOA-dependent glycolysis in HCC cells. RT-qPCR and western blotting were used to detect the expression of ZNF692, KAT5, and ALDOA in HCC cell lines and a normal liver cell line. The influences of transfection-induced alterations in the expression of ZNF692, KAT5, and ALDOA on the functions of HepG2 cells were detected by performing MTT, flow cytometry, Transwell, cell scratch, and colony formation assays, and the levels of glucose and lactate were determined using assay kits. ChIP and luciferase reporter assays were conducted to validate the binding of ZNF692 to the KAT5 promoter, and co-IP assays to detect the interaction between KAT5 and ALDOA and the acetylation of ALDOA. ZNF692, KAT5, and ALDOA were highly expressed in human HCC samples and cell lines, and their expression levels were positively correlated in HCC. ZNF692, ALDOA, or KAT5 knockdown inhibited glycolysis, proliferation, invasion, and migration and promoted apoptosis in HepG2 cells. ZNF692 bound to the KAT5 promoter and promoted its activity. ALDOA acetylation levels were elevated in HCC cell lines. KAT5 bound to ALDOA and catalyzed ALDOA acetylation. ALDOA or KAT5 overexpression in the same time of ZNF692 knockdown, compared to ZNF692 knockdown only, stimulated glycolysis, proliferation, invasion, and migration and reduced apoptosis in HepG2 cells. ZNF692 promotes the acetylation modification and protein expression of ALDOA by catalyzing KAT5 transcription, thereby accelerating glycolysis to drive HCC cell development.

## Introduction

Globally, liver cancer is a commonly diagnosed malignancy and is among the top common causes of cancer death, with 9.5 new cases and 8.7 deaths per 100,000 people (Rumgay et al. 2022). Hepatocellular carcinoma (HCC) is a predominant type of primary liver cancers, which arises from hepatocytes, the main parenchymal cells of the liver (Chidambaranathan-Reghupaty et al. 2021). Hepatitis B/C virus infection and cirrhosis of hepatitis or alcohol origin are leading risk factors for HCC (Josep et al. 2021). HCC is characterized with molecular diversity, and different aetiologies of HCC are associated with distinct mechanisms of hepatocarcinogenesis (Yang et al. 2019). Therefore, molecular signatures are increasingly used for designing targeted therapeutic trials for HCC.

Zinc finger protein 692 (ZNF692), also known as AREBP, is a transcription factor that can be phosphorylated by AMP-activated protein kinase (AMPK), a crucial cellular metabolic sensor (Inoue and Yamauchi 2006). ZNF692 has been shown to promote cell proliferation in cancers such as lung adenocarcinoma, colon adenocarcinoma, and clear cell renal carcinoma (Wang et al. 2023; Xing et al. 2019; Zhang et al. 2017). A recent study reveals upregulated expression of ZNF692 in HCC cell lines (Cai et al. 2023), but its specific role in HCC is not clear. One of the functions of ZNF692 is to suppress the transcription of phosphoenolpyruvate carboxykinase (PCK) and then reduce gluconeogenesis in hepatocytes (Shirai et al. 2011). Gluconeogenesis is a metabolic process in which non-carbohydrate substrates such as lactate are transformed into glucose to meet energy demands under sustained starvation or stress (Yu et al. 2021). There is a reverse of gluconeogenesis, called glycolysis, during which glucose is broken down to pyruvate (Judge and Dodd 2020). Considering the negative effect of ZNF692 on gluconeogenesis, we hypothesized a positive regulatory effect of ZNF692 on the reverse process glycolysis. Importantly, glycolysis is accelarated in tumors and pyruvate is converted into lactate to maintain tumor cell survival (Chen et al. 2021). Therefore, based on our hypothesis, ZNF692 may exert oncogenic effects by promoting glycolysis.

Aldolase A (ALDOA) is an enzyme that cleaves fructose 1,6-bisphosphate into two triosephosphates in the fourth step of glycolysis (Pirovich et al. 2021). ALDOA has been shown to regulate glycolysis in many cancers, including HCC (Lin et al. 2022; Shen et al. 2020; Wang et al. 2022). The GEPIA database shows a positive correlation between the expression of ZNF692 and ALDOA in HCC. Therefore, if ZNF692 promotes glycolysis in HCC, it may achieve this by regulating the glycolytic enzyme ALDOA. Acetylation seems to be an important modification that modulates ALDOA expression and activity in tumor cells (Jiao et al. 2022; Minic et al. 2023). Lysine acetyltransferase 5 (KAT5), also known as Tip60, modulates the activity of key enzymes in gluconeogenesis and glycolysis in HCC cells by acetylation (Lin et al. 2009; Park et al. 2016). However, there is no report on KAT5-mediated acetylation of the glycolytic enzyme ALDOA. Interestingly, the GEPIA database shows that KAT5 expression is positively correlated with both ZNF692 expression and ALDOA expression in HCC. Therefore, ZNF692 may regulate ALDOA in HCC by modulating KAT5-mediated acetylation, thereby promoting glycolysis and tumor growth.

This study analyzed the function of ZNF692 in HCC cells and validated the hypothetical glycolytic axis ZNF692/KAT5/ALDOA. The molecular interactions discovered in this study may be novel targets for the treatment of HCC.

## Materials and methods

### Cell culture and transfection

Human HCC cell lines Huh-7, HepG2, Huh-1, and Hep3B (the Cell Bank of the Chinese Academy of Sciences, Shanghai, China) and human liver epithelial THLE-3 cells (ATCC, USA) were cultured in RPMI1640 (72400120; Gibco, New York, USA) with 10% fetal bovine serum (FBS; 16140071; Gibco) at 37 °C with 5% CO_2_.

The ZNF692 knockdown vector (sh-ZNF692), ALDOA overexpression vector (OE-ALDOA), ALDOA knockdown vector (sh-ALDOA), KAT5 overexpression vector (OE-KAT5), KAT5 knockdown vector (sh-KAT5), and negative controls (sh-NC and OE-NC) were from GeneChem (Shanghai, China). HepG2 cells (3 × 10^5^/dish) were cultured in 60-mm dishes for 24 h in advance. Vectors (3 µg) were incubated with Lipofectamine 2000 reagent (11668019; Invitrogen, CA, USA) in Opti-MEM I reduced serum medium (31985062; Gibco). Cells were incubated in vector-containing media for 48 h.

### MTT assay

After vectors were successfully transfected into HepG2 cells, the cells were cultured in a 96-well plate (3000 cells/well) at 37 °C for 48 h. Then the medium was deserted and the cells were incubated with MTT solution (5 mg/mL, 20 µL/well) for 4 h. After that, 150 µL of DMSO was added to each well to dissolve the formazan under rotation for 10 min. The absorbance at 490 nm of each well was measured with a microplate reader. The cell viability of each experiment group was calculated with the control as the standard (100%).

### Colony formation assay

Cells in the logarithmic growth phase were digested with 0.25% trypsin, suspended in a 10% FBS-containing medium, and seeded at 500 cells/well in 6-well plates, of which each well contained 10 mL of medium prewarmed at 37 °C. The cells were cultured for 2–3 weeks at 37 °C with 5% CO_2_ and saturated humidity until there were colonies visible to the naked eye. The supernatants were deserted and the plate was washed twice with PBS. The cells in each well were fixed with 5 mL of acetic acid-methanol (1:3) for 15 min. Then the fixative was discarded and the cells were dyed with Giemsa stain for 10–30 min. The staining solution was washed away with flowing water and the plate was air-dried. The plate was inverted and overlaid with a transparent grid film. The number of colonies having over ten cells was counted under a microscope (low magnification).

### Scratch assay

Cells were treated with 100 µg/mL mitomycin C (HY-13316; MCE, USA) for 4 h, digested, and resuspended. The cell suspension was transferred to a 12-well plate (1 × 10^5^ cells/well, three wells per group). After the cells grew to 100% confluence, a scratch was made with a 10 µL pipette tip on the bottom of the well. The plate was washed thrice with DPBS (14190250; Gibco) to remove exfoliated cells and loaded with fresh 2% FBS-containing DMEM. At 0 and 24 h, cells in the same field of view were photographed under an Olympus inverted microscope to observe changes in scratch width. Cell migration = (scratch width _0 h_ − scratch width _24 h_)/scratch width _0 h_.

### Transwell assay

Transwell membranes were precoated with matrigel at 37 °C for 2 h. Cells (2 × 10^4^) transfected for 48 h were suspended in a 1% FBS-containing medium and seeded in the upper chamber of Transwell plates (three wells per group), and 0.8 mL of 10% FBS-containing medium was added to the lower chamber. After 48 h, the Transwell insert was taken out and the cells on the upper membrane were removed with a cotton swab. The cells that had passed through the membrane were fixed with methanol for 30 min, stained with 10% Giemsa stain, washed thrice with PBS, and imaged under an inverted microscope.

### Flow cytometry

After digestion, 1 × 10^6^ cells were centrifuged, washed twice with precooled PBS, and resuspended in 100 µL of 1 × binding buffer. After treatment with 5 µL of Annexin V-FITC and 5 µL of PI (BMS500FI-300; eBioscience, USA) in the dark at ambient temperature for 10 min, the cells were added to 400 µL of 1 × binding buffer and the cell apoptosis was analyzed with a flow cytometer within 1 h.

### Glucose and lactate detection

The levels of glucose and lactate in cells were detected with a glucose detection kit (S0201S; Beyotime, Shanghai, China) and lactate dehydrogenase detection kit (C0016; Beyotime) to evaluate the uptake of glucose and production of lactate.

### Chromatin immunoprecipitation (ChIP)

A ChIP kit (26156; Thermo Fisher Scientific, New York, USA) was used. A total of 5 × 10^8^ cells were cultured to be adherent and then incubated with 1% paraformaldehyde at ambient temperature for 10 min. The crosslinking reaction was stopped by adding glycine. The cells were scraped off, centrifuged, and mixed with protease inhibitor-containing lysis buffer by vortexing for 15 s. The chromatin was broken by sonication into fragments of 300–1000 bp. Fifty microliters of supernatant was stored at − 20 °C as input, and 500 µL of supernatant was mixed with 1 mL of hybridization buffer and incubated with 50 pmol of anti-IgG or anti-ZNF692 antibody (1:300, PCRP-ZNF692-1D2; Developmental Studies Hybridoma Bank, Iowa City, IA, USA) at 37 °C for 4 h. Then the samples were kept with washed streptavidin beads under rotation at 37 °C for 30 min and centrifuged. The bead complexes were washed five times and incubated with 50 µL of DNA elution buffer, 100 µg/mL RNase A, and 0.1 U/µL RNase H at 37 °C for 30 min. The samples were added to 10 µL of 5 × loading buffer, boiled at 100 °C for 10 min, and centrifuged. The supernatant was taken for RT-qPCR detection.

### Luciferase reporter assay

The KAT5 promoter sequence and ZNF692 overexpression vector (OE-ZNF692) were both from Genechem. The KAT5 promoter sequence was cloned into pGL3-Basic vector and co-transfected with OE-ZNF692 or sh-ZNF692 into HepG2 cells. After 48 h, Renilla luciferase activity in the cells was detected with a Dual-Glo luciferase reporter assay system (Promega, USA), normalized to the internal control firefly luciferase activity.

### Co-immunoprecipitation (co-IP)

Proteins from cell lysates were incubated with an anti-KAT5 antibody (1:500, ab300521; Abcam, Cambridge, UK), anti-ALDOA antibody (1:300, ab181662; Abcam), or anti-IgG antibody (as an NC; 1:500, ab172730; Abcam) at 4 °C overnight. The immune complexes were incubated with Protein G/A beads under rotation at 4 °C for 3–5 h. The bead-bound complexes were collected by centrifugation at 1000 × *g* and 4 °C for 5 min and washed thrice with washing buffer (50 mM Tris-HCl [pH 7.4], 100 mM NaCl, 5 mM CaCl_2_, 5 mM MgCl_2_, and 0.1% Nonidet P-40). Eluted immune complexes were resuspended in 1 × SDS-PAGE loading buffer, denatured in a metal bath at 100 °C for 5 min, and then loaded on 10% polyacrylamide gels for electrophoresis. After separation, the proteins were transferred onto a PVDF membrane and detected using the western blotting method with an anti-ALDOA antibody (1:2000, ab181662; Abcam) and anti-acetyl Lysine antibody (1:1000, ab190479; Abcam).

### Nude mouse xenograft model

Four-week-old male BALB/c nude mice were purchased from Hunan SJA Laboratory Animal Co., Ltd. (Hunan, China). HepG2 cells stably transfected with sh-ZNF692 or sh-ALDOA and untransfected control HepG2 cells were digested with trypsin and resuspended; 1.0 × 10^7^ cells were subcutaneously injected into the right armpit of nude mice, which were then observed for 25 consecutive days. Tumor volume [length (mm) × width (mm)^2^/2] was measured every five days. The mice were sacrificed for isolation of tumors, which were then assessed, photographed, and used for subsequent experiments.

### Immunohistochemistry staining

Paraffin-embedded sections of the transplanted tumors were dewaxed, incubated with 3% H_2_O_2_ at room temperature for 5–10 min, heated in PBS buffer in a microwave oven for 10–15 min, cooled at room temperature, and soaked in goat serum at room temperature for 15 min. Then the sections were incubated with a Ki67 primary antibody (1:1000, ab15580; Abcam, Cambridge, UK) at 4 °C overnight, followed by incubation with a secondary antibody (1:2000, ab6721; Abcam) at 37 °C for 1 h, nuclear staining with DAPI (D9542; Sigma, Germany) for 3 min, counterstaining with hematoxylin for 2 min, and differentiation with hydrochloric acid alcohol for 2 s. After dehydration and transparentization, the sections were mounted for observation.

### RT-qPCR

Cells were lysed in 1 mL of TRIzol (Thermo Fisher Scientific) to extract total RNA, which was then reverse-transcribed with M-MLV reverse transcriptase and random primers to obtain cDNA. The PCR reaction system was configured and the reaction conditions for each primer were set according to the Premix Ex Taq™II kit (Takara, Tokyo, Japan). PCR experiments were performed using an ABI7500 qPCR instrument (Applied Biosystems, Waltham, MA, USA), with GAPDH as the internal reference. Data was analyzed with the 2^−ΔΔCt^ method (Burja et al. 2019): ΔΔCt = (Ct _target gene_ – Ct _reference gene_) _experiment group_ – (Ct _target gene_ – Ct _reference gene_) _control group_. Table 1 lists the sequences of the PCR primers.

**Table 1:** Primer sequences used in the study

Primers	Sequences (5′-3′)
ALDOA-F	GAGGCGTCCATCAACCTCAA
ALDOA-R	TGCCTATCCTTACCAGGGCT
KAT5-F	TAACACTCCGTTTTCCCCCG
KAT5-R	GGGACCTTTGAGAGACACCG
ZNF692-F	TTTCCAGCCTACCTGCAAGC
ZNF692-R	GTGCTCTTGTCTCATGCCCAC
GAPDH-F	GTGGCTGGCTCAGAAAAAGG
GAPDH-R	GGGGAGATTCAGTGTGGTGG

F, forward primer; R, reverse primer

### Western blotting

The concentration of total protein from lysed cells was determined with a BCA kit (23227; Thermo Fisher Scientific). Protein samples were diluted in 5 × sample buffer, electrophoresed on 12% separating gels for 1.5 h, and transferred onto a membrane, which was then immersed in PBS blocking solution containing 5% (w/v) non-fat powdered milk for 60 min at ambient temperature. After that, the membrane was incubated overnight at 4 °C with anti-ZNF692 (1:1500, ab204595), anti-KAT5 (1:1500, ab300521), anti-ALDOA (1:1500, ab252953), anti-acetyl Lysine (1:1500, ab190479), and anti-GAPDH (1:1500, ab9485) (all from Abcam) antibodies. After washing, the membrane was placed in secondary antibody solution (1:1500, ab150077; Abcam) for 1 h at ambient temperature and imaged using a BioSpectrum system (UVP, USA).

### Statistical analyses

Data analyses were performed using GraphPad Prism 8.0, with all quantitative data presented as mean ± SEM. After Shapiro–Wilk test of normality, data between three and more groups were compared using one-way analysis of variance followed by Bonferroni test, and two sets of data were compared using Student’s t-test. *P* < 0.05 represents statistical significance.

## Results

### ZNF692 and ALDOA accelerate glycolysis and promote HCC cell development

**Fig. 1:**
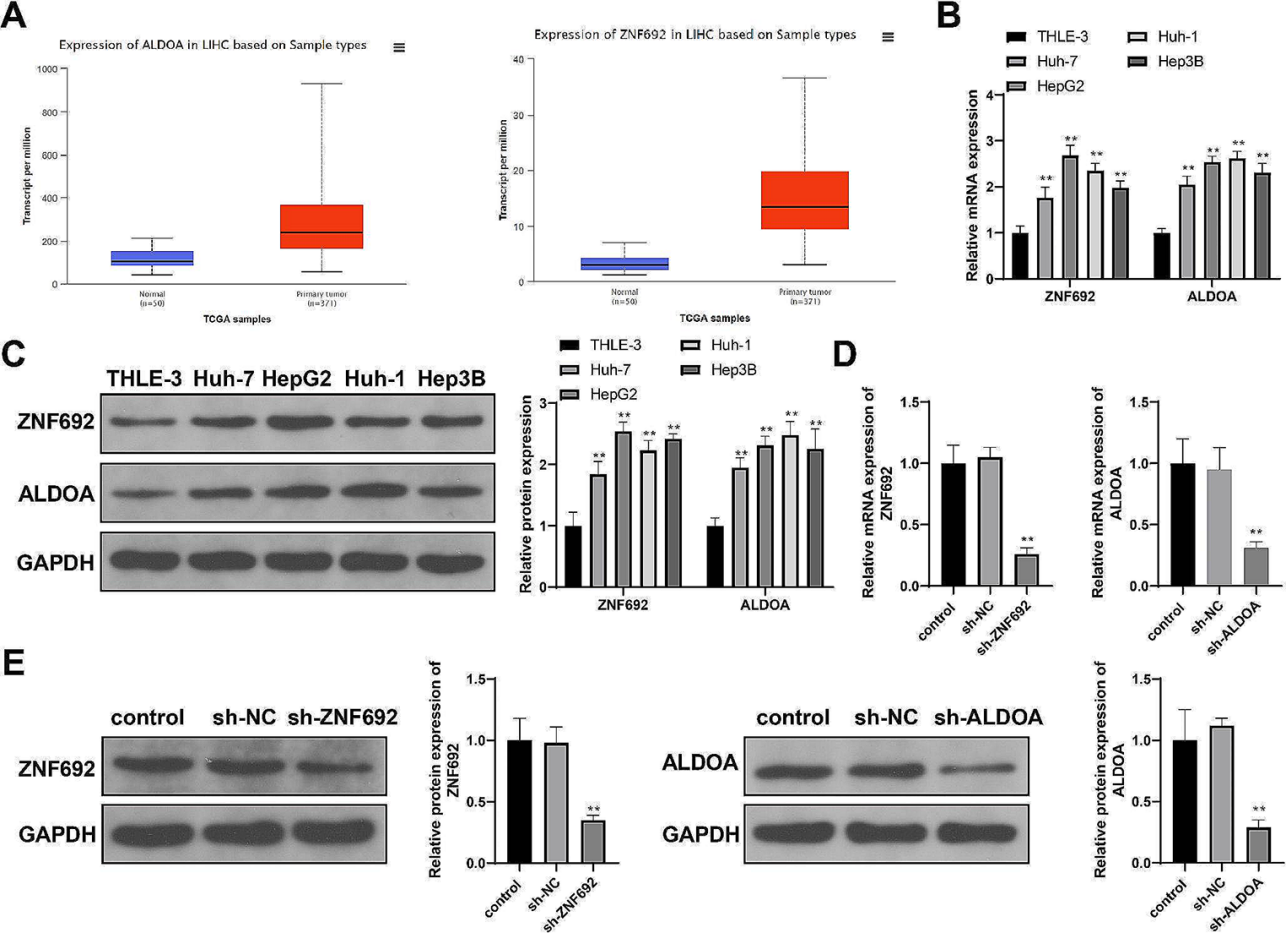
High expression of ZNF692 and ALDOA in HCC. (**A**) ZNF692 and ALDOA expression in TCGA HCC samples. (**B**-**C)** RT-qPCR (B) and western blotting (**C**) detection of ZNF692 and ALDOA expression in HCC cell lines. (**D**-**E**) RT-qPCR (**D**) and western blotting (**E**) detection of ZNF692 expression in HepG2 cells transfected with sh-ZNF692 and ALDOA expression in HepG2 cells transfected with sh-ALDOA. *N* = 3; **P < 0.01 vs. THLE-3 or sh-NC

The TCGA database showed that ZNF692 and ALDOA were highly expressed in HCC (Fig. 1A). Consistently, HCC cell lines (Huh-7, HepG2, Huh-1, and Hep3B) expressed high levels of ZNF692 and ALDOA relative to normal liver epithelial THLE-3 cells (Fig. 1B-C, *P* < 0.01). To determine the functions of ZNF692 and ALDOA in HCC cells, we transfected sh-NC, sh-ZNF692, or sh-ALDOA into HepG2 cells and detected the changes in cell phenotypes. The effect of the transfection was first examined. ZNF692 or ALDOA expression was significantly reduced in cells transfected with sh-ZNF692 or sh-ALDOA, respectively, indicating successful transfection (Fig. 1D-E, *P* < 0.01).

**Fig. 2:**
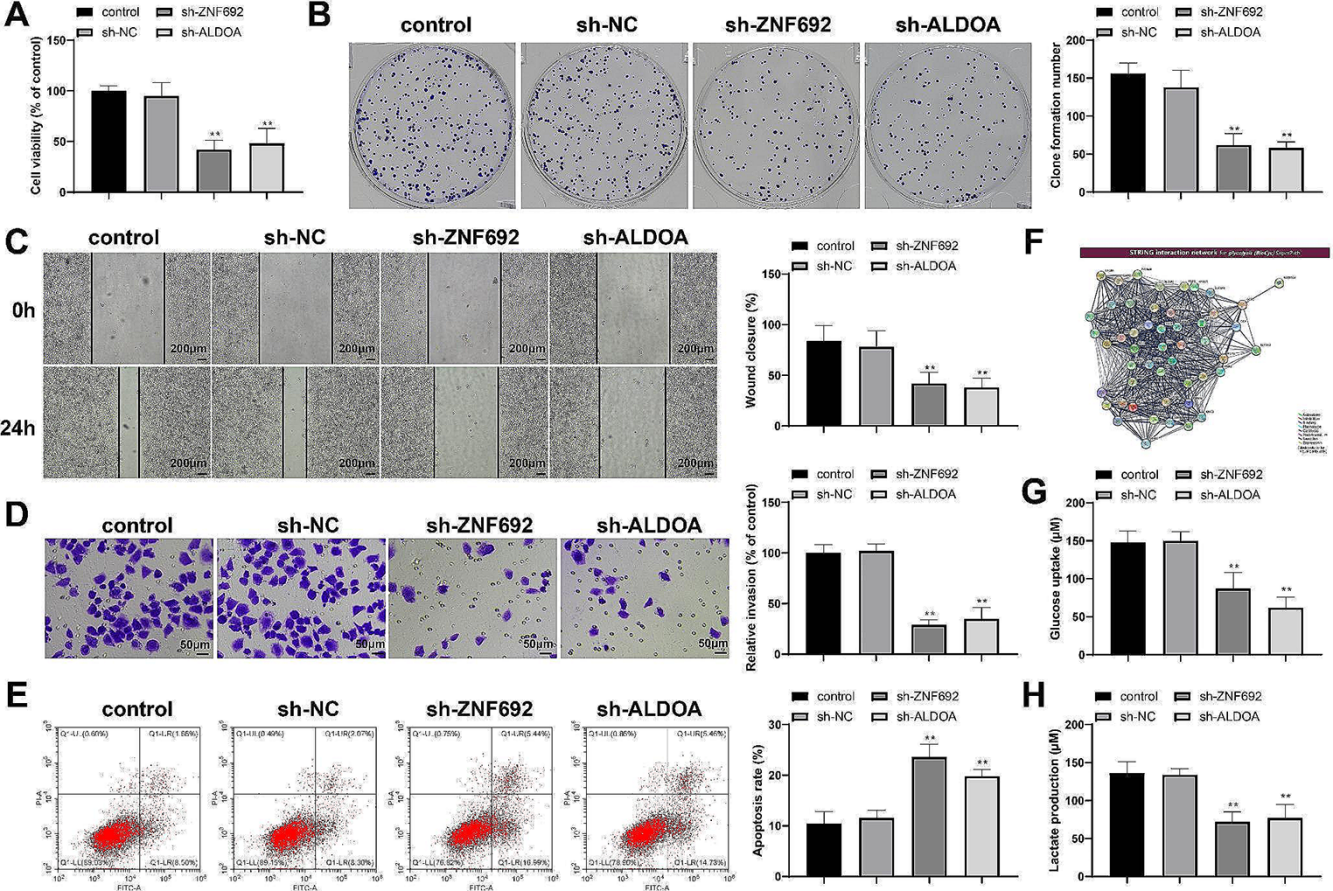
ZNF692 or ALDOA knockdown inhibits proliferation and glycolysis of HCC cells. HepG2 cells were transfected with ZNF692 or ALDOA knockdown vectors: (**A**-**B**) MTT (**A**) and colony formation (**B**) assays were performed to detect cell proliferation; (**C**) scratch assay to detect cell migration; (**D**) Transwell assay to detect cell invasion; and (E) flow cytometry analysis to detect cell apoptosis. (**F**) The PathCards database showed the regulation of ALDOA on glycolysis signaling pathways. (**G**-**H**) Detection kits were used to detect the glucose uptake (**G**) and lactate production (**H**) of HepG2 cells. *N* = 3; **P < 0.01, vs. sh-NC

ZNF692 or ALDOA knockdown reduced proliferation (Fig. 2A, *P* < 0.01), colony formation (Fig. 2B, *P* < 0.01), migration (Fig. 2C, *P* < 0.01), and invasion (Fig. 2D, *P* < 0.01) and increased apoptosis (Fig. 2E, *P* < 0.01) in HepG2 cells. Moreover, the PathCards database (https://pathcards.genecards.org/) showed that ALDOA can directly regulate glycolysis signaling pathways (Fig. 2F). ZNF692 or ALDOA knockdown reduced the glucose uptake and lactate production of HepG2 cells (Fig. 2G-H, *P* < 0.01). These results indicate that ZNF692 and ALDOA potentiate the glycolytic activity and malignant development of HCC cells.

### Knockdown of ZNF692 or ALDOA inhibits HCC growth in vivo

**Fig. 3:**
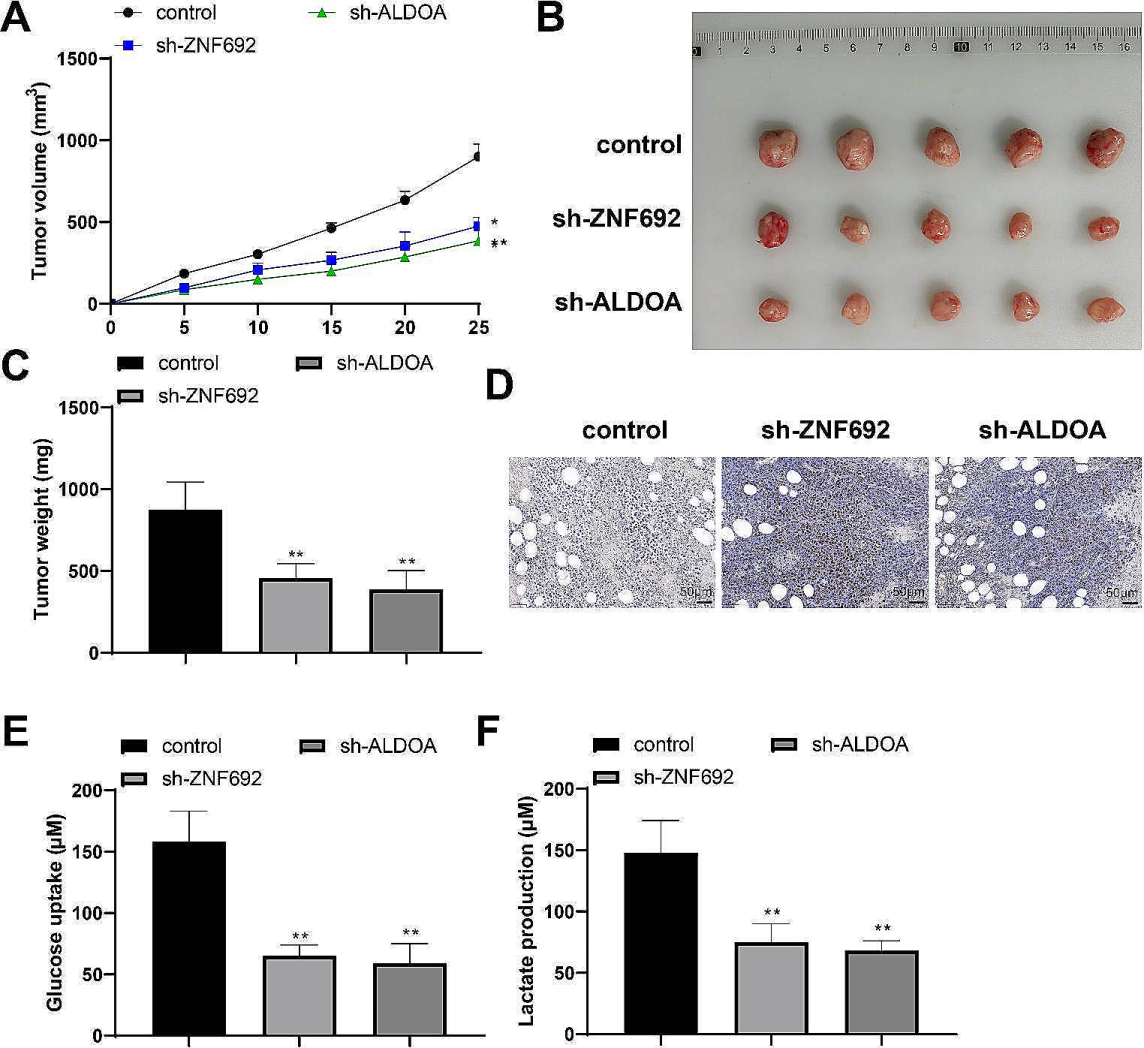
Knockdown of ZNF692 or ALDOA inhibits HCC growth in vivo. A subcutaneous tumor xenograft model was established using nude mice: (**A**) Tumor growth monitoring. (**B**-**C**) The volume (**B**) and weight (**C**) of isolated tumors. (**D**) Immunohistochemistry staining to detect Ki67 expression in tumors. (E-F) The glucose uptake (**E**) and lactate production (**F**) in tumors. *N* = 5; **P < 0.01

To detect the regulatory effects of ZNF692 and ALDOA on HCC growth in vivo, HepG2 cells transfected with sh-ZNF692 or sh-ALDOA were injected subcutaneously into nude mice to establish a tumor xenograft model and then the growth of tumors in each group was observed. Compared with the control group, the sh-ZNF692 and sh-ALDOA groups showed lower tumor growth rates (Fig. 3A, *P* < 0.05). At the end of the experiment, the tumors were isolated and their sizes were measured. The tumor volume and weight of the sh-ZNF692 and sh-ALDOA groups were significantly lower than those of the control group (Fig. 3B-C, *P* < 0.05). Moreover, fewer Ki67-positive cells were detected in the tumors of the sh-ZNF692 and sh-ALDOA groups (Fig. 3D). The uptake of glucose and production of lactate were also reduced in the tumors of the sh-ZNF692 and sh-ALDOA groups (Fig. 3E-F, *P* < 0.05). These data demonstrate the ability of ZNF692 or ALDOA knockdown to inhibit HCC growth and glycolysis in vivo.

### KAT5 drives glycolysis and malignant development of HCC cells

**Fig. 4:**
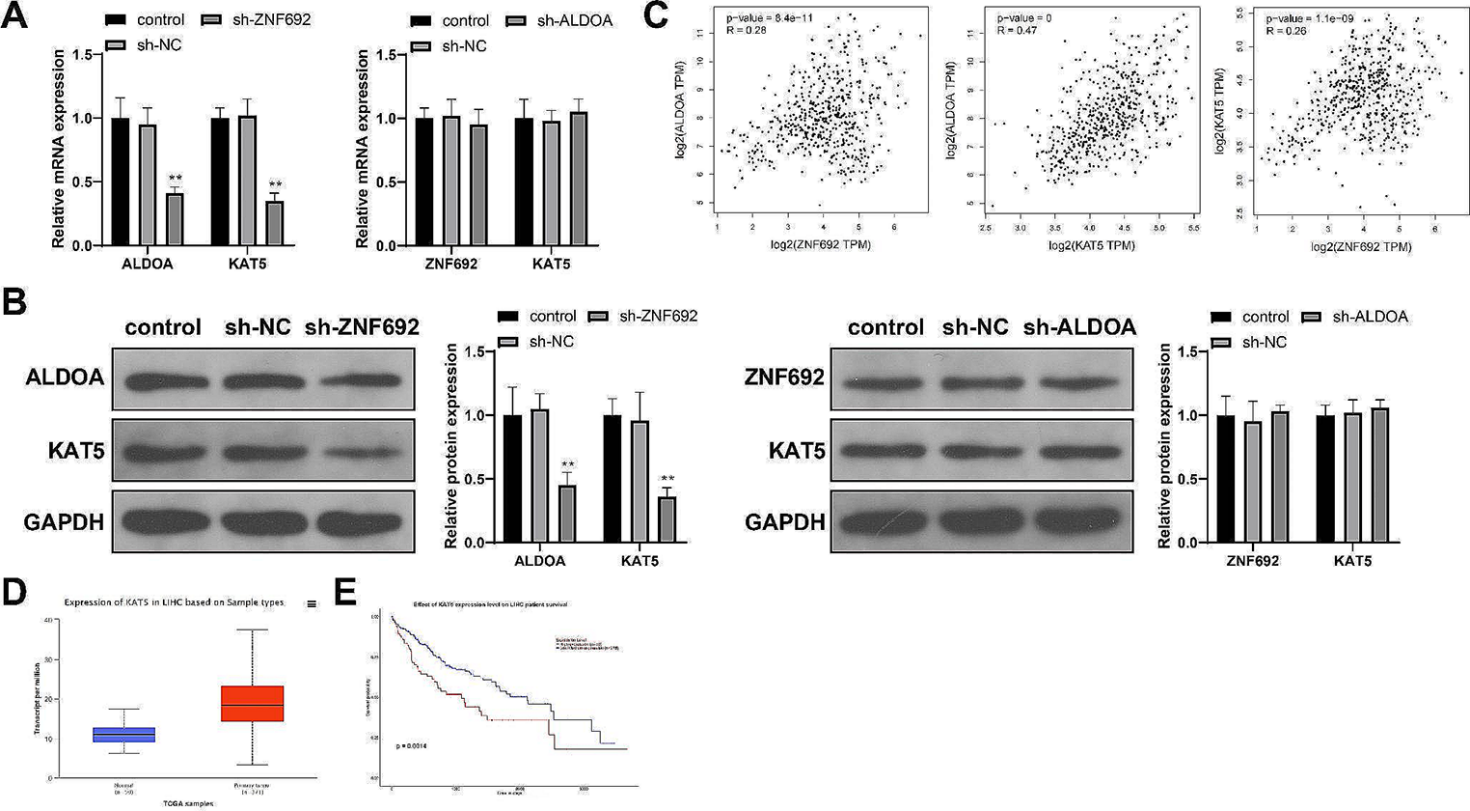
The expression of KAT5 is positively correlated with that of ZNF692 and ALDOA. (**A**-**B**) RT-qPCR (**A**) and western blotting (**B**) detection of ALDOA and KAT5 expression in HepG2 cells transfected with sh-ZNF692 and ZNF692 and KAT5 expression in HepG2 cells transfected with sh-ALDOA. (**C**) The correlations between the expression of ZNF692, ALDOA, and KAT5 in patients with HCC. (**D**) KAT5 expression in TCGA HCC samples. (**E**) The correlation between KAT5 expression and prognosis for patients with HCC. *N* = 3; **P < 0.01, vs. sh-NC

We detected ZNF692 and ALDOA expression in HepG2 cells transfected with sh-ZNF692 or sh-ALDOA and found decreases in ALDOA expression after sh-ZNF692 transfection (Fig. 4A-B, *P* < 0.01) but no significant changes in ZNF692 expression after sh-ALDOA transfection (Fig. 4A-B, *P* > 0.05). These results suggest that ZNF692 is an upstream regulator of ALDOA. The GEPIA database (http://gepia.cancer-pku.cn/) showed there were positive correlations in HCC between ZNF692 and ALDOA expression, KAT5 and ALDOA expression, and KAT5 and ZNF692 expression (Fig. 4C, *P* < 0.01). Moreover, KAT5 was expressed at high levels in TCGA HCC samples (Fig. 4D) and associated with poor prognosis for patients with HCC (Fig. 4E). KAT5 expression was downregulated in HepG2 cells transfected with sh-ZNF692 (Fig. 4A-B, *P* < 0.01) but remained almost unchanged in those transfected with sh-ALDOA (Fig. 4A-B, *P* > 0.05). Therefore, we speculated that KAT5 may participate in the progression of HCC and that ZNF692 may regulate ALDOA via KAT5.

**Fig. 5:**
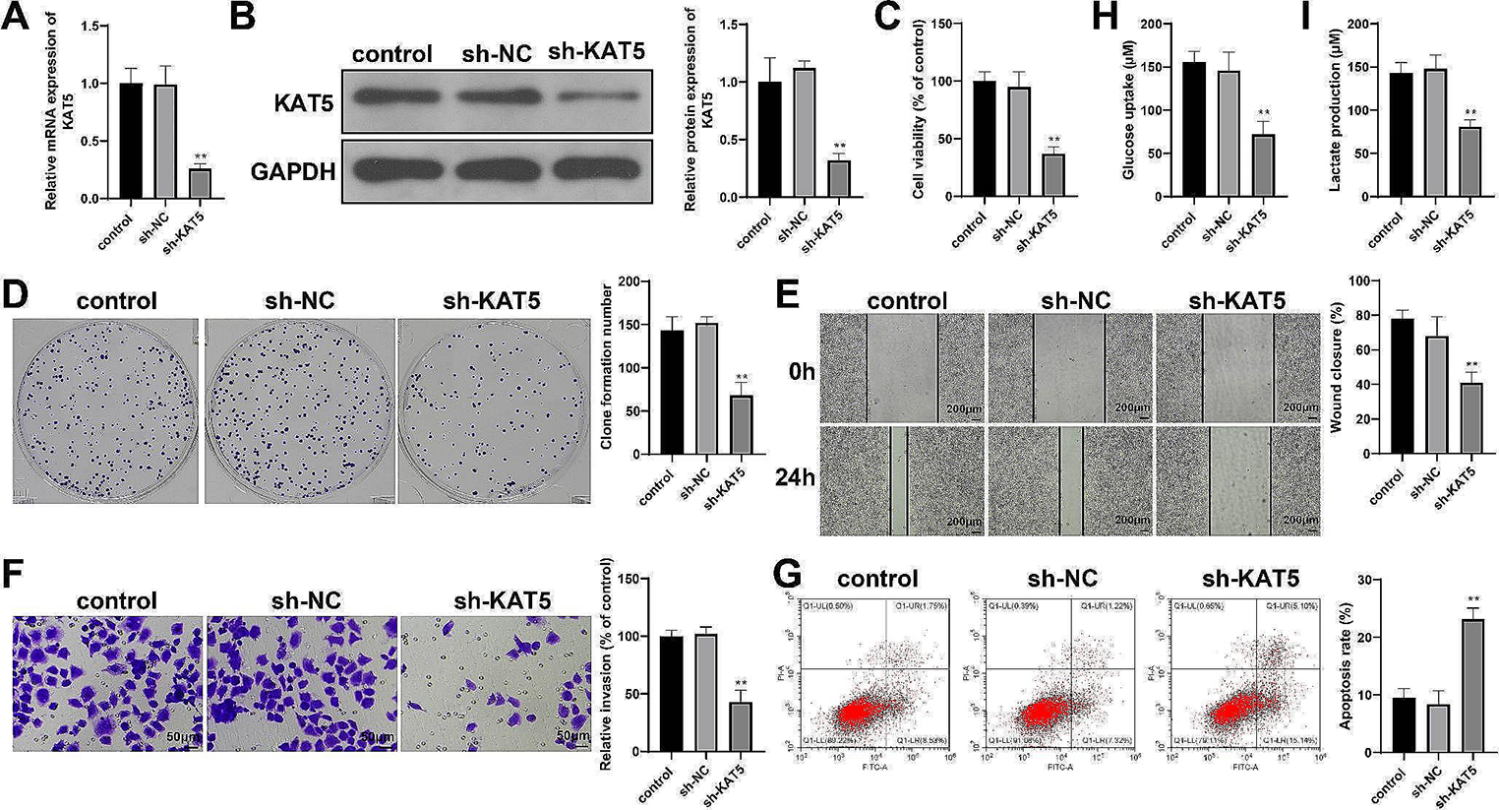
KAT5 knockdown inhibits proliferation and glycolysis of HCC cells. HepG2 cells were transfected with sh-KAT5 or sh-NC: (A-B) RT-qPCR (**A**) and western blotting (**B**) were used to detect KAT5 mRNA and protein expression; (**C**-**D**) MTT (**C**) and colony formation (**D**) assays to detect cell proliferation; (**E**) scratch assay to detect cell migration; (**F**) Transwell assay to detect cell invasion; (**G**) flow cytometry analysis to detect cell apoptosis; and (**H**-**I**) detection kits to detect glucose uptake (**G**) and lactate production (**H**). *N* = 3; **P < 0.01, vs. sh-NC

Next, to investigate the function of KAT5 in HCC cells, we detected the phenotypes of HepG2 cells transfected with sh-NC or sh-KAT5. Compared to sh-NC transfection, sh-KAT5 transfection successfully induced downregulation of KAT5 expression in HepG2 cells (Fig. 5A-B, *P* < 0.01). Moreover, KAT5 knockdown inhibited proliferation (Fig. 5C, *P* < 0.01), colony formation (Fig. 5D, *P* < 0.01), migration (Fig. 5E, *P* < 0.01), invasion (Fig. 5F, *P* < 0.01), glucose uptake (Fig. 5H, *P* < 0.01), and lactate production (Fig. 5I, *P* < 0.01) and stimulated apoptosis (Fig. 5G, *P* < 0.01) in HepG2 cells. These data suggest that KAT5 is conducive to the glycolysis and malignant properties of HCC cells.

### ZNF692 mediates ALDOA expression via KAT5

**Fig. 6:**
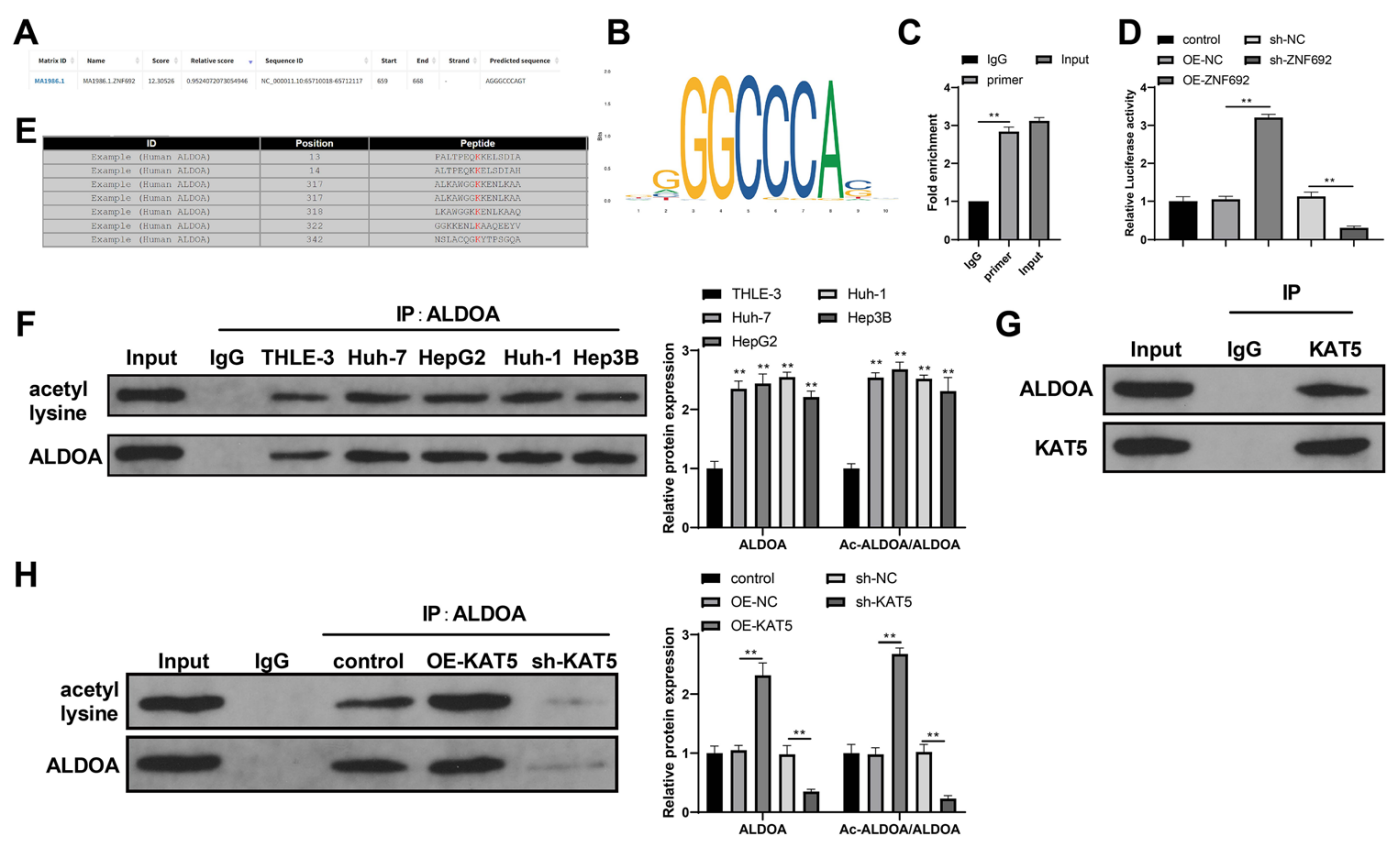
ZNF692 regulates ALDOA acetylation by targeting KAT5. (**A**) The JASPAR database predicted a binding site for ZNF692 in the KAT5 promoter. (**B**) The binding motif of ZNF692. (**C**) ChIP assay was performed to detect the binding of ZNF692 to the KAT5 promoter. (**D**) Luciferase reporter assay was performed to detect the influence of ZNF692 on KAT5 promoter activity. (**E**) The GPS6.0 database predicted several ALDOA acetylation sites. (**F**-**H**) Co-IP experiments were performed to detect ALDOA acetylation levels in HCC cell lines (**F**), the binding of ALDOA to KAT5 in HepG2 cells (**G**), and the effects of alterations in KAT5 expression on ALDOA protein and acetylation levels in HepG2 cells (H). *N* = 3; **P < 0.01

The JASPAR database showed there was a binding site for ZNF692 in the promoter region of KAT5 (Fig. 6A), and the binding motif is shown in Fig. 6B. From this, ZNF692, as a transcription factor, was assumed to regulate the transcription of KAT5. The results of ChIP assay verified that ZNF692 could tightly bind with the KAT5 promoter (Fig. 6C, *P* < 0.01 vs. IgG). Dual-luciferase reporter assay to detect the influence of ZNF692 on KAT5 promoter activity showed that OE-ZNF692 (vs. OE-NC) enhanced luciferase activity while sh-ZNF692 (vs. sh-NC) attenuated luciferase activity in HepG2 cells transfected with the KAT5 promoter-containing luciferase reporter vector (Fig. 6D, *P* < 0.01), indicating that ZNF692 upregulates KAT5 expression by promoting the activity of the KAT5 promoter.

The GPS6.0 database (http://pail.biocuckoo.org/) predicted there were multiple acetylation sites in the amino acid sequence of ALDOA (Fig. 6E), suggesting that KAT5 may regulate ALDOA expression by catalyzing acetylation. Co-IP assay to detect acetylated ALDOA showed that the levels of ALDOA acetylation in HCC cell lines were higher than that in THLE-3 cells (Fig. 6F, *P* < 0.01). Moreover, we used the anti-KAT5 antibody to perform co-IP experiments in HepG2 cells and found ALDOA in the immune precipitates (Fig. 6G). This result demonstrates that KAT5 can bind to ALDOA in HepG2 cells. To confirm the regulation of KAT5 on ALDOA acetylation, we detected ALDOA acetylation levels in HepG2 cells transfected with KAT5-related vectors. OE-KAT5 transfection elevated the levels of ALDOA protein and ALDOA acetylation (vs. OE-NC), whereas sh-KAT5 transfection reduced the levels of ALDOA protein and ALDOA acetylation (vs. sh-NC) (Fig. 6H, *P* < 0.01). This indicates that KAT5 promotes ALDOA acetylation to upregulate ALDOA protein expression.

Collectively, these results demonstrate that ZNF692 promotes KAT5 transcription to catalyze ALDOA acetylation in HepG2 cells, thereby promoting ALDOA expression.

### ZNF692 promotes glycolysis and malignant development of HCC cells via KAT5/ALDOA axis

**Fig. 7:**
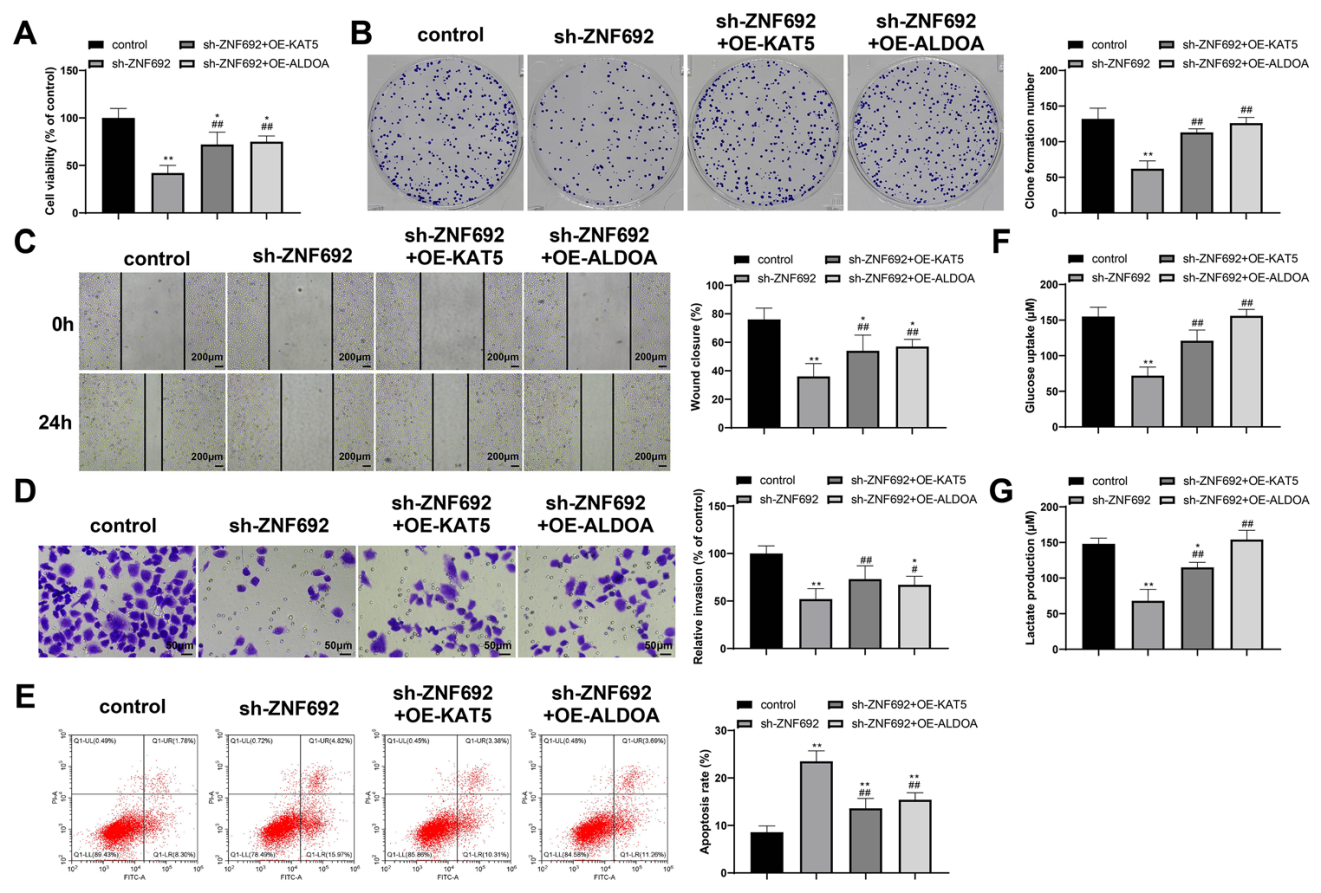
ZNF692 promotes glycolysis and malignant development of HCC cells via KAT5/ALDOA axis. HepG2 cells were transfected with sh-ZNF692, sh-ZNF692 + OE-KAT5, or sh-ZNF692 + OE-ALDOA. (**A**-**B**) MTT (**A**) and colony formation (**B**) assays were used to detect cell proliferation; (**C**) scratch assay to detect cell migration; (**D**) Transwell assay to detect cell invasion; (**E**) flow cytometry analysis to detect cell apoptosis; and (**F**-**G**) detection kits to detect glucose uptake (F) and lactate production (**G**). *N* = 3; *P < 0.05 and **P < 0.01 vs. control; #*P* < 0.05 and ##*P* < 0.01 vs. sh-ZNF692

To examine whether ZNF692 regulates HCC cell phenotypes via the KAT5/ALDOA axis, we transfected HepG2 cells with sh-ZNF692, sh-ZNF692 + OE-KAT5, or sh-ZNF692 + OE-ALDOA. Compared to sh-ZNF692 transfection alone, sh-ZNF692 + OE-KAT5 or sh-ZNF692 + OE-ALDOA co-transfection increased proliferation (Fig. 7A, *P* < 0.01), colony formation (Fig. 7B, *P* < 0.01), migration (Fig. 7C, *P* < 0.01), invasion (Fig. 7D, *P* < 0.05), glucose uptake (Fig. 7F, *P* < 0.01), and lactate production (Fig. 7G, *P* < 0.01) and inhibited apoptosis (Fig. 7E, *P* < 0.01) in HepG2 cells. Altogether, these data imply that ZNF692 promotes the glycolysis and malignant development of HepG2 cells via the KAT5/ALDOA axis.

## Discussion

HCC is a highly fatal malignancy that mostly occurs in individuals with underlying liver disease (Villanueva 2019). Although the main molecular features of HCC have been identified, only 25% of HCCs harbor one druggable target, making it difficult to translate the understanding of the oncogenic drivers and molecular classes into clinical decision-making (Llovet et al. 2022). Therefore, the molecular pathogenesis of HCC still needs exploration to help the emergence of precision oncology strategies. This study discovered that (1) ZNF692 promotes the glycolysis and malignant development of HepG2 cells; (2) ZNF692 and ALDOA promote HCC growth in nude mice; (3) ZNF692 catalyzes the transcription of KAT5; (4) KAT5 acetylates ALDOA and promotes ALDOA protein expression; (5) ZNF692 exerts oncogenic effects in HepG2 cells via the KAT5/ALDOA axis.

ZNF692 plays a carcinogenic role in many cancers. For example, ZNF692 promotes in vitro colon adenocarcinoma (COAD) cell proliferation, migration, and invasion by activating the PI3K/AKT signaling pathway, and high ZNF692 expression is significantly correlated with tumor metastasis in patients with COAD (Xing et al. 2019). MYC binding to the promoter of ZNF692 drives ZNF692 overexpression in clear cell renal carcinoma (ccRCC) cells and in turn, ZNF692 promotes ccRCC proliferation through transcriptional repression of interferon regulatory factor 4 and fms-related receptor tyrosine kinase 4 (Wang et al. 2023). ZNF692 conduces to the malignant phenotypes (proliferation, migration, and invasion) of cervical cancer cells by suppressing p27^kip1^ transcription (Zhu et al. 2019). ZNF692 also contributes to the proliferation, migration, and invasion of lung adenocarcinoma cells and inhibits their apoptosis (Zhang et al. 2017). High expression of ZNF692 is associated with poor overall survival in patients with lung adenocarcinoma (Zhang et al. 2017), osteosarcoma (Sun et al. 2022), or bladder cancer (Wang et al. 2020). Moreover, ZNF692 highly correlates with genes involved in the regulation of metabolic process in lung adenocarcinoma (Zhang et al. 2017). This study found that ZNF692 was highly expressed in TCGA HCC samples and human HCC cell lines. ZNF692 knockdown reduced proliferation, invasion, migration, and glycolysis and incited apoptosis in HepG2 cells. This also limited HCC growth in nude mice. ZNF692 expression was positively correlated with KAT5 expression in HCC. Moreover, ZNF692 promoted KAT5 transcription in HepG2 cells.

KAT5 is an acetyltransferase that shows distinct functions in different cancers (Du et al. 2020; Kim and Lee 2019; Li et al. 2021; Liu et al. 2022). Basically, KAT5 acts as an oncogene in HCC. Vacuolar protein sorting-associated protein 72 homolog drives the proliferation, invasion, and migration of HCC HuH-7 cells by upregulating KAT5 expression to activate the PI3K/AKT signaling pathway (Chen et al. 2022). Depletion of PCK1, a major enzyme of hepatic gluconeogenesis, inhibits KAT5 ubiquitination by increasing its O-GlcNAcylation and subsequently promotes HCC metastasis through KAT5-mediated acetylation of key genes related to epithelial-mesenchymal transition (Liu et al. 2021). Bromodomain-containing 8, negatively regulated by microRNA (miR)-876-3p, promotes proliferation and limits apoptosis in HCC cells probably by increasing KAT5 expression (Yu et al. 2020). LincRNA ZNF337-AS1 promotes KAT5-mediated acetylation of histone H2A.Z to drive HCC growth and metastasis (Yuan et al. 2021). Consistent with the previous findings, high expression of KAT5 was detected in TCGA HCC samples and associated with poor prognosis. KAT5 knockdown suppressed the glycolysis and malignant development of HepG2 cells. Moreover, KAT5 expression was positively correlated with ALDOA expression in HCC.

ALDOA is a key enzyme in glycolysis and an essential driver of metabolic adaptation to hypoxia in HCC (Niu et al. 2021). High expression of ALDOA is associated with unfavorable prognosis for HCC (Tang et al. 2021). Several genes have been discovered to mediate the expression of ALDOA in HCC. Transcription factor MKL1 promotes the expression of PINK1-AS, which in turn increases ALDOA expression by targeting miR-34a-5p, thereby driving glycolysis in HCC cells (Wang et al. 2022). Neuronal PAS domain protein 2, a core regulator of circadian rhythms, upregulates the expression of several glycolytic genes including ALDOA in HCC cells by directly promoting the transcription of hypoxia-inducible factor alpha (Yuan et al. 2020). Acetylated ALDOA is detected in HCC cell-derived small extracellular vesicles (sEVs) and the enzymatic activity of acetylated ALDOA is higher in HCC MDA-MB-231-derived sEVs than in non-tumorigenic MCF10A-derived sEVs (Minic et al. 2023), suggesting the importance of acetylation for the enzymatic activity of ALDOA in HCC. This study found that ALDOA acetylation levels were increased in HCC cell lines. KAT5 promoted ALDOA acetylation and protein levels in HepG2 cells. ALDOA knockdown undermined the proliferation, invasion, migration, and glycolysis of HepG2 cells and suppressed HCC growth in vivo. Moreover, KAT5 or ALDOA overexpression nullified the inhibitory effects of ZNF692 knockdown on the development of HepG2 cells.

In summary, ZNF692 may enhance the malignant phenotype of HCC cells by increasing KAT5 expression to promote ALDOA acetylation and glycolysis. This study first elucidates the function of ZNF692 in HCC cells and ZNF692 may be a novel candidate target for HCC treatment. For the translation into future clinical practice, our data should be further validated in other HCC cell lines, animal models, and human samples.

## Data Availability

The datasets used or analyzed during the current study are available from the corresponding author on reasonable request.
